# Implications for Sentinel Lymph Node Biopsy Omission in Patients with Early-Stage Node-Negative HR+/HER2− Breast Cancer Undergoing Mastectomy

**DOI:** 10.1245/s10434-026-19588-z

**Published:** 2026-04-08

**Authors:** Jennifer H. Chen, Kerollos Nashat Wanis, Mahdia Rahman, Ayushi Gianchandani, Isabelle Bedrosian, Nina Tamirisa, Min Yi, Akshara Raghavendra, Puneet Singh, Mediget Teshome, Susie Sun, Henry Kuerer, Kelly K. Hunt, Funda Meric-Bernstam, Taiwo Adesoye

**Affiliations:** 1 Department of Breast Surgical Oncology, The University of Texas MD Anderson Cancer Center, Houston, TX USA; 2 The University of Texas at Austin, Austin, TX USA; 3 Imperial College London, London, England; 4 Department of Breast Medical Oncology, The University of Texas MD Anderson Cancer Center, Houston, TX 77030 USA; 5 Department of Surgery, Division of Surgical Oncology, UCLA Health Jonsson Comprehensive Cancer Center/David Geffen School of Medicine, Los Angeles, CA USA; 6 Investigational Cancer Therapeutics, The University of Texas MD Anderson Cancer Center, Houston, TX USA

**Keywords:** HR+/HER2− breast cancer, SOUND, INSEMA, Sentinel lymph node biopsy, Axillary ultrasound, Total mastectomy

## Abstract

**Introduction:**

The SOUND and INSEMA trials have demonstrated the non-inferiority of sentinel lymph node (SLN) biopsy (SLNB) omission in patients with hormone receptor-positive (HR+)/human epidermal growth factor receptor 2-negative (HER2−) early-stage breast cancer (BC) with negative axillary ultrasound undergoing breast-conserving therapy (BCT). We evaluated SLNB positivity rates and treatment characteristics among patients undergoing BCT versus mastectomy.

**Methods:**

Patients with cT1N0M0 HR+/HER2− unifocal invasive ductal carcinoma with negative axillary ultrasound undergoing upfront BCT + radiation therapy (RT) or mastectomy +/− RT were included (2010–2023). Clinicopathologic characteristics, treatment, and patient outcomes were compared by surgery type.

**Results:**

Among 1506 patients, the median age was 59 years (interquartile range [IQR] 51–67). In total, 78.2% (1178) underwent BCT and 21.8% (328) underwent mastectomy. The mastectomy cohort was significantly younger and had larger and higher-grade tumors (age 55.7 vs. 60.8; grade 3: 20.1 vs. 12.5%, both *p *< 0.001). Rates of positive SLNs were similar in BCT and mastectomy cohorts (7.6 vs. 8.2%, *p *= 0.684). Among the mastectomy cohort, 10.1% received RT and 28.1% received chemotherapy, with higher rates in patients with positive SLNs (RT: 51.8 vs. 6.31%, chemotherapy: 76.5 vs. 22.5%, both *p* < 0.0001). At a median follow up time of 25.3 months (IQR 13.2–58.9), three (0.2%) had axillary recurrences (two after mastectomy) and seven (0.46%) had distant recurrences (two after mastectomy).

**Conclusions:**

Among patients with cT1N0M0 HR+/HER2− disease and negative AUS who underwent SLNB, early recurrence rates were low regardless of surgery type. Given that nodal status affects the use of RT after mastectomy, further research investigating the omission of SLNB in this cohort is warranted.

**Supplementary Information:**

The online version contains supplementary material available at 10.1245/s10434-026-19588-z.

Sentinel lymph node biopsy (SLNB) has long been the standard for axillary staging in patients with early-stage clinically node negative (cN0) breast cancer (BC). Although SLNB itself is not thought to be therapeutic, it provides essential information to guide the use of adjuvant systemic therapy and radiation therapy (RT). The publication of pivotal surgical de-escalation trials such as SOUND (Sentinel Node versus Observation After Axillary Ultra-Sound)^1^ and INSEMA (Intergroup-Sentinel-Mamma)^2^ have demonstrated the oncologic safety of SLNB omission in selected patients with cN0 status undergoing breast-conserving therapy (BCT) based on axillary ultrasound (AUS). Both studies demonstrated low rates of SLN positivity among patients who underwent nodal evaluation (13.7% in SOUND [8.6% with macrometastatic disease]^1^ and 15% in INSEMA [11.6% with macrometastatic disease]^2^), with no differences in survival outcomes with omission of SLNB.

Of note, both the SOUND and the INSEMA trial included predominantly patients with hormone receptor-positive (HR+)/human epidermal growth factor receptor 2-negative (HER2−) disease and grade 1–2 tumors that were up to 2 cm in size. Importantly, all patients enrolled received BCT with the assumption that whole breast irradiation would adequately treat small-volume nodal metastasis in the axilla, which are typically found by SLNB. Thus, a key limitation of both studies is the omission of patients undergoing mastectomy and the inherent study designs that make it challenging to implement SLNB omission in the mastectomy patient population. Many patients who are candidates for BCT may pursue mastectomy because of genetic predisposition or patient preference.^3^ Furthermore, patients undergoing mastectomy may have low-risk disease, similar to patients in SOUND and INSEMA, with negative clinical and ultrasound axillary evaluations, and yet still undergo routine SLNB despite the low likelihood of nodal involvement. At our institution, we have integrated routine preoperative AUS into clinical practice as a part of our standardized preoperative staging algorithm. This approach allows for more refined preoperative axillary evaluation that enhances our ability to identify patients with node-negative disease with a sensitivity of 86.4% and specificity of 100% when combined with fine-needle aspiration (FNA) of sonographically indeterminate and/or suspicious lymph nodes.^4^

In this study, we leveraged our institutional experience with standardized preoperative AUS to evaluate SLN positivity rates in patients with HR+/HER2− disease undergoing BCT and mastectomy. We evaluated adjuvant treatment implications, including the use of adjuvant systemic therapy and radiotherapy. Our objective was to determine whether the de-escalation framework previously validated in the SOUND and INSEMA studies may be extended to include patients undergoing mastectomy with similar tumor biology in the setting of a negative AUS.

## Methods

### Study Design and Patient Selection

This was a retrospective cohort study conducted at a comprehensive cancer center. We identified patients diagnosed with clinical T1N0M0 BC from January 1, 2010 to December 31, 2023. Eligible patients had unifocal HR+/HER2− invasive ductal carcinoma with a negative baseline AUS and underwent BCT (followed by RT) or mastectomy with SLNB at our institution. Patients were excluded if they had biopsy-proven nodal metastasis, received neoadjuvant systemic therapy, or had recurrent disease and/or previously treated BC. Inclusion criteria for patient selection were limited to patients with clinical T1 HR+/HER2− disease as this was the predominant patient population in the SOUND and INSEMA studies (95.3 and 90.0% T1, 87.8 and 95.2% HR+/HER2−, respectively). The institutional review board at The University of Texas MD Anderson Cancer Center approved this study (protocol 2024-0333).

### Patients and Treatment

Demographic factors, clinicopathologic characteristics, and treatment information were extracted from a prospectively maintained institutional database. Local–regional treatment consisted of SLNB and either BCT or mastectomy with or without RT. All patients underwent SLNB. Based on institutional practice, RT was administered to the primary tumor bed and chest wall, with or without regional nodal irradiation (supraclavicular, infraclavicular, and internal mammary nodal basins). Systemic therapies included adjuvant chemotherapy and/or endocrine therapy. Information on the 21-gene recurrence score (RS) assay was included when available.

### Outcomes

The primary outcomes of interest were the overall rate of SLN positivity and the impact of nodal status on receipt of radiation and systemic therapies. Secondary outcomes of interest included rates of axillary lymph node dissection, incidence of pN2 disease (four or more positive nodes), axillary and distant recurrence at last follow-up, and treatment patterns stratified by surgical approach and RS.

### Statistical Analysis

Descriptive statistics were used to report patient demographics and clinicopathologic characteristics. Student’s t-test and the chi-squared test were used to compare baseline patient differences and treatment characteristics by surgery type (breast conservation vs. mastectomy). Within type of surgery received, treatment characteristics were further compared by SLN positivity and 21-gene RS (≤ 25 vs. > 25). Additional subgroup analysis was performed to identify characteristics associated with receipt of adjuvant RT among patients undergoing mastectomy using multivariable logistic regression analysis. All analyses were performed using Stata (StataCorp, College Station, TX, USA).

## Results

### Patient Demographics and Tumor Characteristics

In total, 1506 patients with cT1N0M0 HR+/HER2− BC were included. The median age at diagnosis was 59 years (IQR 51–67, Table 1). Most patients were White (*n *= 1136 [75.4%]) and postmenopausal (*n *= 1207; [80.1%]) with body mass index ≥ 25 kg/m^2^ (*n *= 988 [65.6%]). Breast-conserving surgery was performed in 78.2% (*n *= 1178) of patients and mastectomy was performed in 21.8% (*n *= 328). Patients in the mastectomy cohort were more likely to be younger (median age 55.7 vs. 60.8 years), premenopausal (30.8 vs. 16.8%), and with BMI < 25 kg/m^2^ (40.2 vs. 22.0%, all *p* < 0.001).

**Table 1:** Baseline characteristics among patients with cT1 N0 M0 hormone receptor-positive/human epidermal growth factor receptor 2-negative breast cancer (2010–2023)

Characteristic	Total (*N *= 1506)	BCT (*n* = 1178)	Mastectomy (*n *= 328)	*p* value
Age	59 (51.3–67.1)	60.8 (53.2–67.8)	55.7 (46.9–64.9)	**< 0.001**
Age group, years				**< 0.001**
<40	54 (3.6)	23 (1.9)	31 (9.4)	
40–49	253 (16.8)	175 (14.9)	78 (23.8)	
50–59	453 (30.1)	358 (30.4)	95 (29.0)	
60–69	500 (33.2)	414 (35.1)	86 (26.2)	
>70	246 (16.3)	208 (17.7)	38 (11.6)	
Race				0.62
White	1136 (75.4)	898 (76.2)	238 (72.6)	
Black	126 (8.4)	94 (8.0)	32 (9.8)	
Asian	83 (5.5)	61 (5.2)	22 (6.7)	
Other	161 (10.7)	125 (10.6)	36 (10.9)	
BMI				**< 0.001**
< 25	391 (26.0)	259 (22.0)	132 (40.2)	
≥ 25	988 (65.6)	812 (69.0)	176 (53.7)	
Unknown	127 (8.4)	107 (9.0)	20 (6.10)	
Menopause				**< 0.001**
Pre	299 (19.9)	198 (16.8)	101 (30.8)	
Post	1207 (80.1)	980 (83.2)	227 (69.2)	
Grade				**< 0.0001**
1	254 (16.9)	203 (17.2)	51 (15.5)	
2	635 (42.2)	481 (40.8)	154 (47.0)	
3	212 (14.1)	146 (12.5)	66 (20.1)	
Unknown	405 (26.8)	348 (29.5)	57 (17.4)	
Pathologic tumor size				**0.002**
Tis	31 (2.1)	14 (1.2)	17 (5.2)	
T1mi	12 (0.8)	4 (0.3)	8 (2.4)	
T1a	132 (8.8)	105(8.9)	27 (8.2)	
T1b	479 (31.8)	391 (33.2)	88 (26.8)	
T1c	713 (47.3)	565 (47.9)	148 (45.1)	
T2	133 (8.8)	97 (8.2)	36 (10.9)	
T3	6 (0.4)	2 (0.2)	4 (1.2)	
SLN positive				0.684
Yes	116 (7.7)	89 (7.6)	27 (8.2)	
No	1390 (92.3)	1089 (92.4)	301 (91.8)	
pN positive				**0.013**
pN0	1347 (89.4)	1070 (90.8)	277 (84.5)	
pN1 (mic)	50 (3.3)	32 (2.7)	18 (5.5)	
pN1	103 (6.8)	74 (6.4)	29 (8.8)	
pN2	5 (0.3)	2 (0.2)	3 (0.9)	
pN3	1 (0.1)	0 (0.0)	1 (0.3)	
Completion ALND				**< 0.001**
No	1481 (98.3)	1175 (99.7)	306 (93.3)	
Yes	25 (1.7)	3 (0.3)	22 (6.7)	

Bold values indicate statistically significant *p* < 0.05

Data are presented as n (%) or median (interquartile range) unless otherwise indicated

ALND, axillary lymph node dissection; BCT, breast-conservation therapy; M, mastectomy (total mastectomy, skin-sparing mastectomy, nipple-sparing mastectomy); BMI, body mass index; SLN, sentinel lymph node

#### Sentinel Lymph Node Positivity

The overall SLN positivity rate was 7.7% (*n *= 116) and did not differ by the type of surgery received (7.6% for breast conservation vs. 8.2% for mastectomy, *p* = 0.68). A total of 171 patients had indeterminate findings on preoperative axillary ultrasound and subsequently underwent FNA to rule out the presence of nodal metastases. The SLN positivity rate in this cohort was slightly higher, at 8.2%, compared to 7.6% in patients who did not undergo FNA.

Overall final pathologic nodal burden was distributed as follows: 89.4% (*n* = 1347) pN0, 3.3% (*n *= 50) pN1 (mic), 6.8% (*n* = 103) pN1, 0.3% (*n *= 5) pN2, and 0.07% (*n* = 1) pN3 (Table 1). Rate of ALND was 1.7% (*n* = 25), with the majority performed in patients undergoing mastectomy compared with breast conservation (6.7 vs. 0.3%, *p *< 0.001) (Table 1). On final pathologic nodal evaluation, patients undergoing mastectomy were more likely to have positive nodes than those undergoing breast conservation (pN1–3: 15.5 vs. 9.3%, *p *= 0.013).

#### Treatments Received

Adjuvant treatments included chemotherapy in 21.8% of patients (*n *= 329), endocrine therapy in 71.8% (*n* = 1082), and RT in 80.4% (*n *= 1211). Patients who underwent mastectomy were more likely to receive chemotherapy (28.1 vs. 20.1%, *p* = 0.002) and endocrine therapy (77.8 vs. 70.2%, *p *= 0.023) compared to patients who underwent breast-conserving surgery (Table 2). Among patients who underwent mastectomy, those with SLN-positive disease were more likely to receive chemotherapy than SLN-negative patients (76.5 vs. 22.5%, *p *= 0.0001). A total of 493 patients had 21-gene RS available. Of these, 23.3% (*n* = 115) underwent mastectomy and 76.6% (*n *= 378) underwent BCT. Among postmenopausal patients with positive SLNs and RS ≤ 25, chemotherapy was used in 16.6% (2/12) after BCT and 0% (0/4) after mastectomy. When RS was ≥ 25, chemotherapy use was 60% (27/45) after BCT and 85.7% (12/14) after mastectomy.

**Table 2:** Treatment characteristics by surgery type

Characteristic	Total (*N* = 1506)	BCT (*N* = 1178)	Mastectomy (*N* = 328)	*p* value
Chemotherapy				**0.002**
No	1149 (76.3)	915 (77.7)	234 (71.3)	
Yes	329 (21.8)	237 (20.1)	92 (28.1)	
Unknown	28 (1.9)	26 (2.2)	2 (0.6)	
Endocrine therapy				**0.023**
No	374 (24.8)	308 (26.1)	66 (20.1)	
Yes	1082 (71.8)	827 (70.2)	255 (77.8)	
Unknown	50 (3.3)	43 (3.7)	7 (2.1)	
Radiation therapy				
No	295 (19.6)	0 (0.0)	295 (89.9)	**< 0.0001**
Yes	1211 (80.4)	1178 (100)	33 (10.1)	

Bold values indicate statistically significant *p* < 0.05

Data are presented as n (%) unless otherwise indicated

BCT, breast-conserving therapy

Given that we only included patients receiving BCT (breast-conserving surgery plus RT), all patients undergoing breast-conserving surgery received adjuvant RT, whereas only 10.1% of patients in the mastectomy cohort received RT. Compared with patients who did not receive RT, those who underwent mastectomy and received RT were more likely to be younger (*p *= 0.006), have larger tumor size (p<0.0001) with positive sentinel node (*p *< 0.0001) and undergo ALND (33.3 vs. 3.7%, *p* < 0.001) (Supplementary Table S1). On multivariable logistic regression analysis, patients with positive SLNs and pN1 status were more likely to receive adjuvant RT than those with negative SLNs and pN0 status, respectively (hazard ratio 8.6; 95% confidence interval [CI] 1.2–62.0, *p* = 0.03 and hazard ratio 31.4; 95% CI 4.8–203.5, *p *< 0.001, respectively) (Supplementary Table S2). Additionally, patients aged 40–49 years were less likely to receive adjuvant RT than those aged < 40 years (hazard ratio 0.65; 95% CI 0.01–0.65, *p* = 0.02).

### Summary of Events

At a median follow-up time of 25.3 months (IQR 13.2–58.9), the overall recurrence rate was 1.5% (*n* = 22) (Table 3). The majority of recurrences were local–regional recurrences (156[8.2%]), including 12 in the ipsilateral breast and three in the axilla. Five (22.7%) were distant, and two (9.1%) involved both local–regional and distant sites. Among patients undergoing mastectomy, two had axillary recurrence, one had distant recurrence, and one had both local–regional and distant recurrence. No deaths were recorded for the study cohort on last follow-up. Further details on tumor characteristics, axillary management, and treatments received for recurrence events are described under Table 3.

**Table 3:** Summary of local–regional and distant recurrences

Characteristic	Total (*N *= 22)	BCT (*N* = 10)	Mastectomy (*N *= 12)
Recurrence type			
Local–regional	15 (68.2)	5 (50.0)	10 (83.3)
Ipsilateral breast	12 (54.5)	4 (40.0)	8 (66.7)
Axillary	3 (13.6)	1 (10)	2 (16.7)
Distant	5 (22.7)	4 (40.0)	1 (8.3)
Both	2 (9.1)	1 (10.0)	1 (8.3)
Grade			
1	1 (4.6)	1 (10.0)	0 (0)
2	9 (40.9)	5 (50.0)	4 (33.3)
3	8 (36.4)	5 (40.0)	4 (33.3)
Unknown	4 (18.1)	0 (0)	4 (33.3)
Pathologic tumor size			
T1mi	0 (0)	0 (0)	0 (0)
T1a	1 (4.5)	1 (10.0)	0 (0)
T1b	4 (18.2)	2 (20.0)	2 (16.7)
T1c	17 (77.3)	7 (70.0)	10 (83.3)
SLN positive			
Yes	3 (13.6)	9 (90.0)	2 (16.7)
No	19 (86.4)	1 (10.0)	10 (83.3)
pN positive			
pN0	15 (68.2)	8 (80.0)	7 (58.3)
pN1 (mic)	4 (18.2)	2 (20.0)	2 (16.7)
pN1	3 (13.6)	0 (0)	3 (25.0)
pN2	0 (0)	0 (0)	0 (0)
pN3	0 (0)	0 (0)	0 (0)
ALND			
No	21 (95.5)	10 (100)	11 (91.7)
Yes	1 (4.5)	0 (0)	1 (8.3)
Chemotherapy			
No	12 (54.5)	8 (80.0)	4 (33.3)
Yes	10 (45.5)	2 (20.0)	8 (66.7)
Endocrine therapy			
No	7 (31.8)	2 (20.0)	5 (41.7)
Yes	15 (68.2)	8 (80.0)	7 (58.3)
Radiation therapy			
No	7 (31.8)	0 (0.0)	7 (58.3)
Yes	15 (68.2)	10 (100.0)	5 (41.7)

ALND, axillary lymph node dissection; BCT, breast-conserving therapy; SLN, sentinel lymph node

## Discussion

This study demonstrates that, in a cohort of patients with cT1N0M0, HR+/HER2- invasive ductal carcinoma and negative preoperative AUS, the SLN positivity rate was low, at 7.7%, regardless of the type of surgical resection performed for the primary breast tumor. Only 0.46% of patients had pN2 disease. Among postmenopausal patients with node-positive disease, receipt of chemotherapy appeared to be driven by RS, regardless of surgery type. In contrast, receipt of RT among patients who underwent mastectomy was influenced by nodal status. These findings suggest that the low SLN positivity rates in patients undergoing mastectomy may support consideration of SLNB omission. Omission of SLNB may potentially improve patient-reported outcomes by avoiding an additional axillary procedure and its associated risks of seroma, lymphedema, and arm symptoms, similar to results reported in the INSEMA trial among patients undergoing breast-conserving surgery. In addition, SLNB requires additional resource utilization such as tracer injection, pathologic processing, and operative time when the likelihood of nodal involvement is very low. In these settings, decisions regarding RT may require multidisciplinary discussion to account for individual patient and disease factors.

Our institution’s consistent use of AUS as a routine pre-operative staging modality provides a high level of confidence in excluding patients with higher burden of nodal disease, which offers a unique perspective in applying the findings of de-escalation trials such as SOUND and INSEMA to patients undergoing mastectomy. In a patient cohort with tumor characteristics and biology comparable to those enrolled in the SOUND and INSEMA trials, we observed a lower rate of occult nodal disease on SLNB after AUS (7.7 vs. the 13.7% reported in SOUND or the 15% in INSEMA).^1,2^ This rate falls below the commonly cited FNR threshold for SLNB, which is often considered acceptable up to 10%.^5–7^ Though most of the patients in our cohort did not undergo ALND, the rate of ≥ pN2 disease was low, further emphasizing the low likelihood of high nodal burden in patients with cN0 BC based on a negative AUS. These findings were true regardless of surgical approach in patients carefully selected based on tumor size and HR+/HER2- status. This finding underscores the importance of incorporating HR and HER2 status into axillary management decisions.

Adjuvant systemic treatment recommendations for patients with early-stage HR+/HER2- cT1N0 BC are largely driven by genomic assays, especially among postmenopausal patients. In this contemporary cohort of patients with available RS information, we found that among postmenopausal patients with HR+/HER2− BC with node-positive disease and RS ≤ 25, chemotherapy use was low although sample sizes in our cohort were small (0% [0/4] in mastectomy, 16.6% [2/12] in BCT). In contrast, most of the patients with a RS >25 received chemotherapy regardless of surgical approach or nodal status. These findings suggest a shift towards prioritizing genomic risk over traditional anatomic features such as nodal status in guiding therapy in this population. The use of chemotherapy and/or CDK4/6 inhibitors is recommended in patients with pN2 disease irrespective of genomic assay score,^9^ but this likelihood is low in patients with a negative AUS, as demonstrated in this study and others.^1,2^ Additionally, the NATALEE trial evaluated the CDK 4/6 inhibitor ribociclib and demonstrated benefit in a broader population, including patients with high-risk node-negative disease. As systemic therapy decisions increasingly rely on tumor biology rather than nodal status, these data support investigating de-escalation strategies, including omission of SLNB in appropriately selected patients undergoing mastectomy.

Although nodal information obtained from axillary staging may not significantly influence chemotherapy decisions in postmenopausal patients with HR+/HER2- disease, it may still affect RT recommendations, particularly for those undergoing mastectomy. Postmastectomy RT (PMRT) in the setting of node-positive disease is consistent with current guidelines. As such, node-positive patients undergoing mastectomy were significantly more likely to receive PMRT in this study than those in the node-negative cohort, per the standard of care. Furthermore, although SLN-positivity rates were similar between patients undergoing mastectomy versus BCT, those undergoing mastectomy were more likely to have additional positive nodes detected as they were more likely to undergo ALND. PMRT to the chest wall with or without comprehensive regional nodal irradiation is recommended for patients at high risk for recurrence, such as patients with tumor size >5 cm or node-positive disease.^9^ This is based on the AMAROS ^10^ and, more recently, SENOMAC^11^ trials supporting omission of ALND in patients with low nodal burden receiving regional nodal irradiation, even among patients undergoing mastectomy. However, although these studies demonstrate decreased morbidity with radiotherapy compared with ALND, radiotherapy may still have important implications for breast reconstruction, including higher complication rates and less favorable cosmesis in patients who undergo mastectomy.^12^ Emerging data support a more individualized approach to radiotherapy based on tumor biology. The feasibility of RT omission has been demonstrated in biologically favorable, HR+/HER2- disease in trials such as IDEA ^13^ and LUMINA,^14^ both of which incorporated clinical and molecular risk profiles but required pathologic nodal assessment in selecting optimal patient populations for therapy de-escalation. The ongoing TAILOR RT (NCT03488693)^15^ trial is evaluating radiotherapy omission in patients with HR+/HER2- disease with limited nodal involvement (one to three nodes) and low genomic risk, specifically those with a RS <18 following BCT or mastectomy. Data from this trial may inform a paradigm shift towards radiotherapy de-escalation based on biological risk in patients with early-stage BC.

Our results should be interpreted in the setting of several limitations. This is a retrospective analysis at a single institution where AUS is routinely performed. As such, findings may not be generalizable to centers where AUS is selectively performed and where operator experience and interpretive expertise may differ. Inherent selection bias is present because of the study’s retrospective design. For instance, patients in the mastectomy cohort were more likely to receive chemotherapy and endocrine therapy than were those undergoing BCS, likely because of their younger age and higher-grade disease rather than nodal status alone. However, these findings should be considered exploratory because of the small sample sizes of patients who received adjuvant therapy such as PMRT. Given that this study spanned a 14-year period, we were unable to control for any variations in systemic therapy regimens in response to landmark studies such as TAILORx and RxPONDER. In addition, we reported a shorter median follow-up time of 25.3 months versus 69.6 months and 73.6 months reported in SOUND and INSEMA, respectively. Given the propensity for late disease recurrence in HR+ tumors, a longer period of follow-up will be important to fully assess oncologic outcomes. Our observed pathologic nodal burden in this study cohort was dependent on the extent of axillary surgery performed as only a minority of patients underwent ALND. However, rates of pN1-3 disease were similarly low among the SLNB only versus SLNB with conversion to ALND cohort. Finally, not all postmenopausal patients in our study cohort had genomic testing with 21-gene RS, likely because of the study timeframe. As such, RS results were not uniformly available across the cohort, limiting our ability to fully evaluate the relationships between RS, treatment decisions, and outcomes in all eligible patients.

## Conclusion

We observed low rates of SLN involvement among patients with HR+/HER2- disease with a negative AUS undergoing mastectomy. These findings suggest that axillary surgery de-escalation may be appropriate for select patients undergoing mastectomy with tumor biology similar to that of patients enrolled in the SOUND and INSEMA trials, supporting prospective evaluation of axillary surgery de-escalation in this patient population. However, the continued influence of nodal status on radiotherapy decisions underscores the need for improved tools to guide local–regional therapy in the absence of nodal staging. As systemic therapy decisions increasingly rely on tumor biology rather than anatomic staging, future studies should focus on refining radiation treatment through multidisciplinary and biomarker-informed approaches to optimize outcomes while minimizing overtreatment.

## Supplementary Information

Below is the link to the electronic supplementary material.

Supplementary file1 (DOCX 17 KB)
