# Supplementary material for: Implications for Sentinel Lymph Node Biopsy Omission in Patients with Early-Stage Node-Negative HR+/HER2− Breast Cancer Undergoing Mastectomy
Source: Ann Surg Oncol. 2026 Apr 8;33(7):6414–21. doi: 10.1245/s10434-026-19588-z (PMC13242406; doi:10.1245/s10434-026-19588-z)
Supplement: Supplementary file 1 — Supplementary file1 (DOCX 17 KB) [file 10434_2026_19588_MOESM1_ESM.docx]

SUPPLEMENTARY MATERIALS

**Supplementary Table S1. Characteristics of Patients Undergoing Mastectomy, Stratified by Receipt of Adjuvant RT**

| **Characteristic** | **No RT (N=295)** | **Received RT**  **(N=33)** | **p-value** |
| --- | --- | --- | --- |
| Age group |  |  | **0.006** |
| <40 | 22 (7.5) | 9 (27.3) |  |
| 40-49 | 71 (24.1) | 7 (21.2 ) |  |
| 50-59 | 89 (30.2) | 6 (18.2) |  |
| 60-69 | 79 (26.8) | 7 (21.2 ) |  |
| >70 | 34 (11.5) | 4 (12.1) |  |
| Race |  |  | 0.228 |
| White | 210 (71.2) | 28(84.8) |  |
| Black | 32 (10.8 ) | 0 (0.0) |  |
| Asian | 21 (7.1 ) | 1(3.0) |  |
| Other | 32 (10.8) | 4 (12.1) |  |
| BMI |  |  | 0.749 |
| < 25 | 119 (40.3) | 13 (39.4) |  |
| ≥ 25 | 159 (53.9) | 17 (51.54) |  |
| Unknown | 17 (5.8) | 3 (9.1) |  |
| Menopause |  |  | **0.02** |
| Pre | 85 (28.8) | 16 (48.5) |  |
| Post | 210 (71.2) | 17 (51.5) |  |
| Grade |  |  | 0.05 |
| 1 | 50 (17.0) | 1 (3.0) |  |
| 2 | 140 (47.5) | 14 (42.4) |  |
| 3 | 58 (19.7) | 8 (24.2) |  |
| Unknown | 47 (16.0) | 10 (30.3) |  |
| Pathologic tumor size |  |  | **<0.0001** |
| Tis | 16 (5.4) | 1 (3.0) |  |
| T1mi | 6 (2.0) | 2 (6.1) |  |
| T1a | 25 (8.5) | 2 (6.1) |  |
| T1b | 84 (28.5) | 4 (12.1) |  |
| T1c | 135 (45.8) | 13 (39.4) |  |
| T2 | 28 (9.5) | 8 (24.2) |  |
| T3 | 1 (0.3) | 3 (9.1) |  |
| SLN positive |  |  | **<0.0001** |
| No | 282 (95.6) | 19 (57.6) |  |
| Yes | 13 (4.4) | 14 (42.4) |  |
| pN positive |  |  | **<0.0001** |
| pN0 | 267 (90.5) | 10 (30.3) |  |
| pN1 (mic) | 12 (4.1) | 6 (18.2) |  |
| pN1 | 14 (4.7) | 15 (45.5) |  |
| pN2 | 2 (0.7) | 1 (3.0) |  |
| pN3 | 0 (0.0) | 1 (3.0) |  |
| Completion ALND |  |  | **<0.0001** |
| No | 284 (96.3) | 22 (66.7) |  |
| Yes | 11 (3.7) | 11 (33.3) |  |
| Oncotype score |  |  | 0.358 |
| ≤ 25 | 92 (31.2) | 9 (27.3) |  |
| > 25 | 14 (4.7) | 0 (0.0) |  |
| Unknown | 189 (64.1) | 24 (72.7) |  |

**Supplementary Table S2. Multivariable Logistic Regression Model – Predictors of Adjuvant Radiotherapy Receipt Among Patients Undergoing Mastectomy**

| **Variable** | **Odds Ratio** | **95% CI** | **p-value** |
| --- | --- | --- | --- |
| Age group |  |  |  |
| <40 | *Ref* |  |  |
| 40-49 | 0.65 | 0.01-0.65 | **0.020** |
| 50-59 | 0.79 | 0.01-1.12 | 0.061 |
| 60-69 | 0.74 | 0.04-1.25 | 0.072 |
| >70 | 0.42 | 0.02-7.37 | 0.547 |
| Race |  |  |  |
| White | *Ref* |  |  |
| Non-White | 0.35 | 0.05-2.21 | 0.263 |
| BMI |  |  |  |
| < 25 | *Ref* |  |  |
| ≥ 25 | 0.82 | 0.19-3.41 | 0.786 |
| Unknown | 2.67 | 0.21-34.34 | 0.450 |
| Menopause |  |  |  |
| Pre | *Ref* |  |  |
| Post | 2.52 | 0.19-32.61 | 0.478 |
| Grade |  |  |  |
| 1 | *Ref* |  |  |
| 2 | 5.28 | 0.33-84.31 | 0.239 |
| 3 | 13.44 | 0.76-237.77 | 0.076 |
| Unknown | 13.87 | 0.59-328.86 | 0.103 |
| Pathologic tumor size |  |  |  |
| Tis | *Ref* |  |  |
| T1mi | - |  |  |
| T1a | 0.46 | 0.01-15.93 | 0.665 |
| T1b | 0.29 | 0.01-5.72 | 0.416 |
| T1c | 1.55 | 0.11-2.65 | 0.198 |
| T2 | 0.52 | 0.03-9.97 | 0.664 |
| T3 | - | - | **-** |
| SLN positive |  |  |  |
| No | *Ref* |  |  |
| Yes | 8.57 | 1.18-61.98 | **0.033** |
| pN positive |  |  |  |
| pN0 | *Ref* |  |  |
| pN1 (mic) | 3.74 | 0.29-47.09 | 0.308 |
| pN1 | 31.4 | 4.84-203.54 | **<0.001** |
| pN2 | - |  |  |
| pN3 | - |  |  |
